# Clinical characteristics and risk factor analysis of recipients with multidrug-resistant bacterial bloodstream infections after liver transplantation: a single-centre retrospective study

**DOI:** 10.1080/20523211.2024.2390072

**Published:** 2024-08-20

**Authors:** Chuanlin Chen, Qinghua Guan, Desheng Li, Bo Sheng, Zhenyu Zhang, Yongfang Hu

**Affiliations:** a School of Clinical Medicine, Qinghai University, Xining, People’s Republic of China; b Liver ICU, Beijing Tsinghua Changgung Hospital, School of Clinical Medicine, Tsinghua University, Beijing, People’s Republic of China; c Department of Clinical Pharmacy, Beijing Tsinghua Changgung Hospital, School of Clinical Medicine, Tsinghua University, Beijing, People’s Republic of China

**Keywords:** Organ transplantation, liver transplantation, postoperative complications, blood stream infections, multidrug-resistant bacterial

## Abstract

**Background:**

The clinical characteristics and associated risk factors for recipients who experience multidrug-resistant organism (MDRO) bloodstream infections after liver transplantation are poorly understood. This study aimed to analyse the clinical characteristics and epidemiology of pathogenic bacteria and identify associated risk factors in patients who underwent MDRO after liver transplantation.

**Method:**

We retrospectively collected data on recipients who developed bloodstream infections after liver transplantation between 2018 and 2023. Recipients were divided into MDRO and non-MDRO groups based on blood culture results. We explored the risk factors for MDRO bloodstream infections post-transplantation and summarised the clinical features, pathogen epidemiology, and prognosis. A multivariate logistic regression analysis was conducted to identify significant risk factors.

**Results:**

A total of 463 liver transplant recipients were studied, and 73 developed blood infections. There were 29 MDRO cases. The mean duration of the episodes was 26 days (range: 1–474 days). Among these patients, 22 (30.1%) developed blood infections without fever (temperature < 37.3°C), and 33 patients (45.2%) had a white blood cell count between 4 and 10 × 10⁹/L. Among the 108 positive blood cultures, 29 genera were detected, predominantly gram-negative bacilli (*n* = 64, 58.2%). The detection rate for multidrug-resistant bacilli was 31.8% (35/110), with the abdomen being the most common site of origin (21.3%). Factors such as a history of preoperative intensive care unit (ICU) hospitalisation (*p* < 0.001) and a preoperative international normalised ratio (INR) > 2 (*p* < 0.048) were identified as risk factors in multivariate regression analysis.

**Conclusion:**

Multidrug-resistant bacterial bloodstream infections after liver transplantation tend to occur early in the postoperative period (<30 days) and are associated with high mortality and a lack of specific clinical manifestations. A history of preoperative intensive care unit (ICU) hospitalisation and an international normalised ratio (INR) > 2 may be risk factors for multidrug-resistant bacterial bloodstream infections after liver transplantation.

## Introduction

Liver transplantation (LT) is a critical intervention for a variety of end-stage liver diseases, offering a potential lifeline for affected patients (Green, 2013). Although advances in surgical methods and perioperative care have markedly improved survival rates, LT continues to be plagued by a high incidence of postoperative infections, a critical factor in recipient mortality (Al-Hasan et al., 2009; Linares et al., 2009). Bloodstream infections (BSIs) pose a particular threat, affecting 19% to 40% of transplant recipients and leading to a substantial postoperative mortality rate between 24% and 52% (S. I. Kim et al., 2009; Santos et al., 2016).

A growing concern in this field is the increase in multidrug-resistant organism (MDRO) infections, especially among LT recipients, who are increasingly vulnerable to gram-negative bacilli (Bert et al., 2010; Kritikos & Manuel, 2016). Alarmingly, resistance is observed in approximately 19% of gram-negative bacilli infections treated with broad-spectrum antibiotics (Hand & Patel, 2016). Infections caused by extended-spectrum beta-lactamase (ESBL)-producing gram-negative bacteria after transplantation are associated with significantly higher mortality rates than non-ESBL-producing infections (Kaye et al., 2001; Melzer & Petersen, 2007). This is reflected in the stark contrast in the 1-year survival rate after transplantation, which is up to 90% in recipients without MDRO infections, decreases to 67% in patients with non-MDRO infections, and decreases to 29% in patient with carbapenem-resistant *Klebsiella pneumoniae* infections, highlighting the profound impact of MDRO (Kalpoe et al., 2012).

Despite advances in understanding microbial epidemiology and risk factors for BSIs after LT, most related research has been limited to single-center studies. This limitation leads to variations in findings due to differences in donor characteristics, hospital conditions, antimicrobial approaches, and regional bacterial patterns. Furthermore, the susceptibility of LT recipients to MDRO bloodstream infections, coupled with often subtle clinical symptoms, remains insufficiently addressed in the current literature. Consequently, there is a compelling need for more comprehensive research to reassess the clinical characteristics and manageable risk factors associated with MDROs in LT recipients. This study aimed to analyse the clinical characteristics and epidemiology of pathogenic bacteria and identify associated risk factors in patients who underwent MDRO after LT.

## Method

### Ethics approval

This study was approved by the Ethics Review Committee of Beijing Tsinghua Changgeng Hospital (approval number 24206-4-01, approval date 20 March 2024). Patient consent was not required as this was a retrospective study.

### Study design

**Figure 1.**
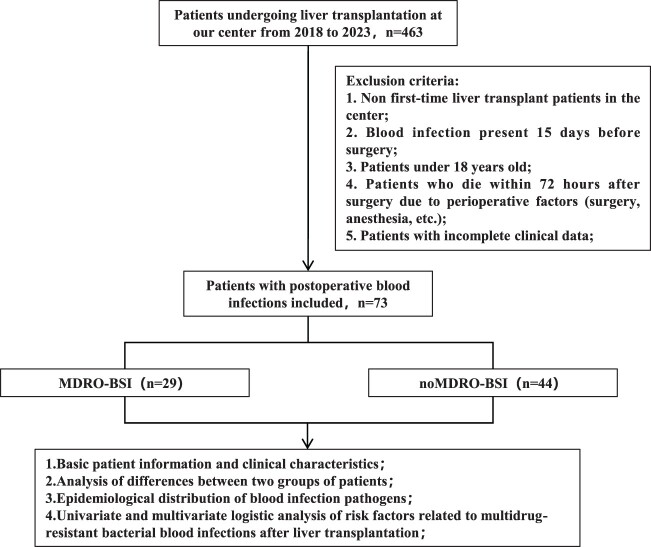
Flowchart of the study.

This retrospective study analyzed data from patients who underwent LT in the liver intensive care unit (ICU) of Beijing Tsinghua Changgung Hospital from January 2018 to December 2023. Based on blood culture results, patients were divided into MDRO and non-MDRO groups (Figure 1).

### Inclusion and exclusion criteria

The inclusion criteria were patients who underwent LT in the liver ICU of Beijing Tsinghua Changgung Hospital from January 2018 to December 2023 and had a diagnosis of bloodstream infections post-LT with clear culture evidence. The exclusion criteria included patients under 18 years old, those who underwent combined liver and kidney transplantation, individuals with re-transplantations, those with incomplete medical records, and recipients with bloodstream infections within 15 days before LT.

### Data collection and immunosuppressive regimen

Laboratory and microbiological data were obtained from the hospital's Department of Medical Laboratory database, while patient data were collected from hospital case files. After liver transplantation, a triple immunosuppressive regimen consisting of tacrolimus (FK506), mycophenolate mofetil (MMF), and glucocorticoids is routinely administered.

### Determination of risk factors

A comprehensive review of the relevant literature was conducted to identify possible risk factors associated with LT (Al-Hasan et al., 2009; Hand & Patel, 2016; Kalpoe et al., 2012; Kaye et al., 2001; S. I. Kim et al., 2009; Y. K. Kim et al., 2002; Kritikos & Manuel, 2016; Liu et al., 2022; Melzer & Petersen, 2007; Park et al., 2020; Santos et al., 2016). These risk factors were categorised into three distinct groups: preoperative, intraoperative, and postoperative parameters. The patients’ demographic data, such as age, sex, and indications for surgery, were analysed. The case history component focused on clinical manifestations, including fever and chills. Various laboratory parameters, including the prothrombin time (PT), international normalised ratio (INR), total bilirubin, albumin, creatinine, potassium, ammonia, and neutrophil ratio (NEUT%), were evaluated. Furthermore, infection-related data, including the identification of pathogenic organisms, infection duration and site, and drug resistance distribution pattern, were examined.

### Related definitions

According to the diagnostic criteria of the China Ministry of Health and the National Healthcare Safety Network (NHSN) of the United States (Garner et al., 1988; ‘Diagnostic Criteria for Hospital-acquired Infections (Trial),’ 2003), a bloodstream infection is identified when a pathogenic bacterium is isolated from a blood sample during hospitalisation, accompanied by one or more of the following: (1) a temperature >38°C or <36°C with chills; (2) invasive portals of pathogens or migration lesions; (3) evident systemic symptoms of infection and toxicity without clear infection foci; and (4) systolic blood pressure <90 mmHg or a decrease of >40 mmHg from baseline. Each bloodstream infection was considered a separate event if two or more microorganisms were isolated at different times if the same genus reappeared 15 days after the initial infection, or if the blood cultures became negative and then became positive again.

Polymicrobial events were counted in cases where two or more isolates grew from the same culture set, except when contaminants were considered. Infections by microorganisms typically found on the skin (*Corynebacterium diphtheriae*-like, *Bacillus* spp., *Propionibacterium* spp., *Micrococcus* spp., or coagulase-negative *Staphylococcus spp*.) require the isolation of at least two blood cultures and evidence of hospital-acquired infection.

Primary bloodstream infection is defined as a bloodstream infection in which no apparent source of infection is identified at the time of diagnosis, and secondary bloodstream infection occurs concurrently with an infection at another site, where the pathogens in the blood culture are common causative organisms of the infection at the other site, suggesting that the bloodstream infection originated at that site (Timsit et al., 2020). Catheter-associated bloodstream infection is diagnosed in patients with an indwelling intravascular device where at least one blood culture drawn from a peripheral vein is positive, along with clinical symptoms of infection (Fang, 2008).

### Bacterial identification and drug susceptibility test

The blood culture procedure was conducted strictly with the established clinical microbiology specifications for blood culture operations (Long & Koyfman, 2016). Blood specimens were cultured using the BacT/Alert 3D automatic blood culture system. The sample was transferred to a blood plate for further culture when an alarm was received for a positive culture. The bacteria isolated from these cultures were identified using the VITEK-2 Compact automatic bacterial identification system and API bacterial identification strips. Drug susceptibility testing was conducted using paper diffusion or dilution methods. The results were interpreted using the 2018 American Clinical Laboratory Standards Institute (CLSI) standards.

### Statistical analysis

The data were analysed using R software, version 4.3. Continuous variables that followed a normal distribution are expressed as the mean ± standard deviation (±SD) and were analysed using the t test. Nonnormally distributed data were evaluated using the two-independent-sample rank-sum test. The χ^2^ test or Fisher's exact probability test was applied for categorical data to compare group differences. Variables with a *p* value < 0.05 in the univariate analysis were included in the multivariate logistic regression analysis. This inclusion was guided by clinical expert opinion, a comprehensive consideration of factor interactions, control and reduction of confounding factors, and the improvement of the internal validity. *P* values, odds ratios (ORs), and 95% confidence intervals (CIs) were calculated for identified risk factors for infection. A *p* value < 0.05 was considered statistically significant. Sample size calculation was not performed as the study was limited by the transplant cases.

## Results

### Patient demographic and clinical characteristics

During the study period, 463 patients underwent LT, and 73 met the inclusion criteria (Figure 1). Among them, 29 patients (39.7%) developed postoperative bloodstream infections with MDROs, while 44 (60.3%) had bloodstream infections without MDROs. Multiple blood infections (≥ 2 instances) were observed in 22 patients. The mean time for the first occurrence of blood infection was 26 days, and 87.7% (64/73) of the patients experienced infections in the early postoperative period (within 30 days).

Regarding clinical symptoms, 22 patients (30.1%, 22/73) did not experience fever (temperature < 37.3°C): 8 (27.6%, 8/29) in the MDRO group and 14 (31.8%, 14/44) in the non-MDRO group. White blood cell (WBC) counts at the onset of blood infection ranged from 4 to 10 × 10^9^ /L in 10 (34.5%, 10/29) and 23 (31.5%, 23/73) patients, respectively. Compared to the non-MDRO group, the MDRO group exhibited significantly higher median levels of C-reactive protein (CRP) (57.47 vs. 16.05, *p* = 0.001). A higher proportion of patients in the MDRO group had a history of preoperative ICU hospitalisation (72.4% vs. 4.6%, *p* < 0.001), ten or more hospitalisations in the year before surgery (27.6% vs. 0%, *p* < 0.001), antibiotic use within 30 days before surgery (79.3% vs. 25%, *p* < 0.001), INR levels ≥ 2 (57.1% vs. 11.4%, *p* < 0.001), and creatinine ≥ 120 mmol/L (72.4% vs. 31.8%, *p* < 0.001). Detailed comparisons of demographic and clinical indicators between the two groups are provided in Table 1.

**Table 1.** The baseline information of 73 recipients with bloodstream infections.

Variables	Total (*n* = 73)	noMDRO BSI (*n* = 44)	MDRO BSI (*n* = 29)	*p*
Sex (male), *n* (%)	53 (72.60)	33 (75.00)	20 (68.97)	0.572
Age (years) ≥ 70, *n* (%)	55.00 (47.00, 63.00)	56.50 (44.75, 64.00)	54.00 (49.00, 62.00)	0.924
BMI, Mean ± SD	23.15 ± 3.77	23.21 ± 3.80	23.07 ± 3.78	0.877
Comorbidities, *n* (%)				0.117
Diabetes	9 (12.33)	8 (18.18)	1 (3.45)	
Hypertension	10 (13.70)	5 (11.36)	5 (17.24)	
Hypertension and diabetes	4 (5.48)	1 (2.27)	3 (10.34)	
Primary disease for LT, *n* (%)				0.331
Acute liver failure	6 (8.22)	5 (11.36)	1 (3.45)	
Alcoholic cirrhosis	9 (12.33)	6 (13.64)	3 (10.34)	
Autoimmune hepatitis	2 (2.74)	2 (4.55)	0 (0.00)	
Decompensation period of hepatitis B cirrhosis	28 (38.36)	18 (40.91)	10 (34.48)	
Hepatic echinococcosis	4 (5.48)	3 (6.82)	1 (3.45)	
Hepatocellular malignant tumour	17 (23.29)	8 (18.18)	9 (31.03)	
Other	7 (9.59)	2 (4.55)	5 (17.24)	
Operation, *n* (%)				0.160
Autologous liver transplantation	2 (2.74)	1 (2.27)	1 (3.45)	
Classic Orthotopic Liver Transplantation	41 (56.16)	21 (47.73)	20 (68.97)	
Improved piggyback liver transplantation	26 (35.62)	18 (40.91)	8 (27.59)	
Split liver transplantation	4 (5.48)	4 (9.09)	0 (0.00)	
Blood concentration of tacrolimus (ng/mL), M (Q₁, Q₃)	7.43 (6.40, 8.33)	7.21 (6.25, 8.05)	7.66 (6.62, 8.49)	0.272
Ascites, *n* (%)	41 (56.16)	25 (56.82)	16 (55.17)	0.890
Hepatic encephalopathy, *n* (%)	13 (17.81)	10 (22.73)	3 (10.34)	0.176
MELD score ≥ 70, *n* (%)	39(53.42)	26(59.09)	13(44.83)	0.232
Chil-puge score, *n* (%)				0.547
A	12(16.44)	6(13.64)	6(20.69)	
B	28(38.36)	16(36.36)	12(41.38)	
C	33(45.21)	22(50.00)	11(37.93)	
Inflammatory indications				
PCT, M (Q₁, Q₃)	5.87 (1.17, 18.80)	3.95 (0.71, 21.72)	7.26 (3.53, 16.80)	0.169
C-reactive protein, M (Q₁, Q₃)	31.00 (12.58, 71.00)	16.05 (8.75, 59.25)	57.47 (21.72, 123.20)	0.001
Lac, M (Q₁, Q₃)	1.80 (1.30, 2.90)	1.70 (1.30, 2.32)	2.00 (1.20, 4.50)	0.132
WBC, M (Q₁, Q₃)	7.28 (4.35, 11.16)	6.76 (4.43, 10.64)	9.09 (4.06, 12.59)	0.579
NLR, M (Q₁, Q₃)	24.39 (13.91, 40.43)	24.93 (15.97, 37.41)	23.23 (13.44, 42.87)	0.960
Preoperative ICU stay history, *n* (%)	23 (31.51)	2 (4.55)	21 (72.41)	<.001
Preoperative history of open abdominal surgery, *n* (%)	24 (32.88)	11 (25.00)	13 (44.83)	0.078
Hospitalisation frequency in 1 year before surgery ≥ 10, *n* (%)	8 (10.96)	0 (0.00)	8 (27.59)	<.001
Plasma exchange, *n* (%)	12 (16.44)	4 (9.09)	8 (27.59)	0.078
Antibacterial drug use within 30 days before LT, *n* (%)	34 (46.58)	11 (25.00)	23 (79.31)	<.001
Time of operationdayu ≥ 8 h, *n* (%)	65 (89.04)	40 (90.91)	25 (86.21)	0.805
The intraoperative blood loss ≥ 2L, *n* (%)	10 (13.7)	5 (11.36)	5 (17.24)	0.714
The intraoperative Urine output mL, M (Q₁, Q₃)	1120 (790–1970)	1495 (798–2319)	950 (780–1700)	0.128
Biliary complications				0.204
Bile leakage	6 (8.22)	2 (4.55)	4 (13.79)	
Narrow	12 (16.44)	6 (13.64)	6 (20.69)	
Intestinal leakage, *n* (%)	9 (12.33)	1 (2.27)	8 (27.59)	0.004
Abdominal paracentesis, *n* (%)	29 (39.73)	10 (22.73)	19 (65.52)	<.001
Transplantation or open laparotomy again, *n* (%)	17 (23.29)	8 (18.18)	9 (31.03)	0.204
Splenectomy, *n* (%)	4 (5.48)	0 (0.00)	4 (13.79)	0.045
Acute rejection reaction, *n* (%)	10 (13.7)	5 (11.36)	5 (17.24)	0.714
CRRT, *n* (%)	25 (34.25)	12 (27.27)	13 (44.83)	0.122
International normalised ratio ≥ 2, *n* (%)	20 (27.4)	5 (11.36)	15 (51.72)	<.001
Total bilirubin (μmol/L) ≥ 122, *n* (%)	36 (49.32)	25 (56.82)	11 (37.93)	0.114
Albumin (g/L) < 35, *n* (%)	44 (60.27)	29 (65.91)	15 (51.72)	0.226
Creatinine (mmol/L) ≥ 120, *n* (%)	35 (47.95)	14 (31.82)	21 (72.41)	<.001
Length of ICU stay days, M (Q₁, Q₃)	7.0 (5.0–12.0)	6.5 (5.0–10.0)	7.0 (6.0–12.0)	0.172
Ventilator support time (h), M (Q₁, Q₃)	10.0 (6.0–24.0)	11.0 (6.8–23.3)	8.0 (5.0–48.0)	0.826
Total length of hospital stay (day), M (Q₁, Q₃)	28.0 (22.0–43.0)	27.0 (19.0–43.8)	29.0 (24.0–38.0)	0.520
28 d death, *n* (%)	11 (15.07)	2 (4.55)	9 (31.03)	0.006
90 d death, *n* (%)	13 (17.81)	2 (4.55)	11 (37.93)	<.001

BMI: Body Mass Index; MDRO: Multidrug Resistant Organism; PCT: Procalcitonin; Lac: Lactic acid; WBC: White blood cell count; NLR: Neutrophil-to-lymphocyte ratio; CRRT: Continuous renal replacement therapy

### Impact of MDRO infections on postoperative outcomes

Compared to the non-MDRO group, the MDRO group had significantly longer ICU stays (21 days vs. 2 days, *p* < 0.001). The incidence of postoperative bowel leakage was higher in the MDRO group (27.6% vs. 2.3%, *p* = 0.004), as was the frequency of abdominal paracentesis procedures (65.5% vs. 22.7%, *p* < 0.001). This group also had higher 28-day (31.0% vs. 4.6%, *p* = 0.006) and 90-day mortality rates (37.9% vs. 4.6%, *p* < 0.001). The details are shown in Table 1.

### The distribution of blood infection pathogens

A total of 29 genera were identified in 108 positive blood cultures. Of these, 10 were attributed to catheter colonisation, and 98 were attributed to blood infections. Two cultures had complex polymicrobial blood infections: one with *E. faecalis* and *Candida albicans* and the other with *E. faecalis* and *Staphylococcus epidermidis*. The predominant pathogens were gram-negative bacilli (58.2%, 64/110), followed by gram-positive cocci (32.7%, 36/110) and fungi (9.1%, 10/110). The details are shown in Table 2.

**Table 2.** Distribution of pathogens isolated from blood samples of liver transplant recipients.

Pathogen	The number of pathogen detections (110)	Percentage (%)
**Gram negative bacilli**	**64**	**58**.**2**
*Klebsiella pneumoniae*	35	31.8
*Enterobacter cloacae*	3	2.7
*Escherichia coli*	3	2.7
*Enterobacter aerogen*	1	1.8
*Acinetobacter baumannii*	10	9.1
*Burkholderia*	3	2.7
*Stenotrophomonas maltophilia*	2	2.8
*Pseudomonas aeruginosa*	1	1.8
*Corynebacterium striatum*	1	1.8
Salmonella Group D	1	1.8
*Citrobacter freudii*	1	1.8
*Serratia marcescens*	1	1.8
*Acinetobacter genus*	1	1.8
*Pantoea* spp	1	1.8
**Gram positive cocci**	**36**	**32.7**
*Enterococcus faecium*	16	14.5
*Staphylococcus epidermidis*	13	11.8
*Staphylococcus aureus*	1	1.8
*Staphylococcus capitis*	2	2.8
*Enterococcus durans*	1	1.8
*Staphylococcus hominis*	1	1.8
*Staphylococcus capris*	1	1.8
*Oral Streptococci*	1	1.8
**Fungus**	**10**	**9.1**
*Smooth Candida*	2	2.8
*Candida albicans*	2	2.8
*Candida parapsilosis*	2	2.8
*Tropical Candida*	1	1.8
*Cryptococcus neoformans*	1	1.8
*Fusarium genus*	1	1.8
*Saccharomyces cerevisiae*	1	1.8

The detection rate for drug-resistant bacteria was 54.5% (60/110), with MDROs accounting for 31.8% (35/110). Table 3 presents the detailed distribution of drug-resistant pathogens.

**Table 3.** Distribution of resistance rates of pathogenic bacteria.

Pathogen	The number of pathogen detections (110)	Percentage (%)
Drug-resistant gram-negative bacteria	38	34.5
*Klebsiella pneumoniae* CRE	25	22.2
*Klebsiella pneumoniae*/*Escherichia coli* ESBLs	4	3.6
*Enterobacter cloacae* CRE	1	0.9
*Acinetobacter baumannii* CRAB	8	7.3
Drug-resistant gram-positive bacteria	22	20
*Staphylococcus epidermidis* MRS	11	10
*Enterococcus faecalis* HLAR	4	3.6
*Staphylococcus aureus* MRSA	1	2.7
*Staphylococcus mansoni* MRS	2	1.8
*Staphylococcus caprae* MRS	1	0.9
*Staphylococcus capitis* MRS	3	0.9

**Figure 2.**
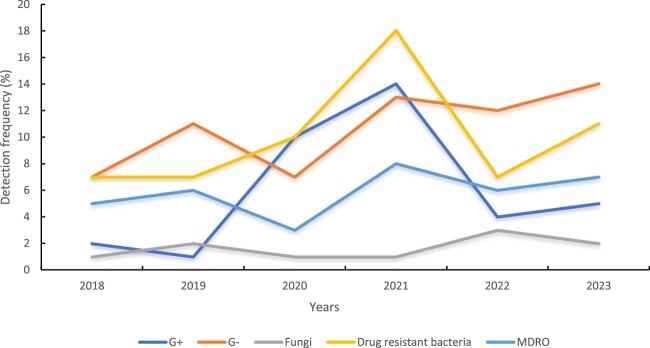
Annual epidemiological trends of pathogenic bacteria.

The origins of the BSI pathogens were unknown (45.9%, 45/98), and there were secondary abdominal infections (23.5%, 23/98), catheter-associated infections (22.4%, 22/98), secondary catheter infections (5.1%, 5/98), and secondary lung infections (3.1%, 3/98). The annual epidemiological trends of pathogenic bacteria are shown in Figure 2.

### Univariate and multivariate logistic analyses of risk factors associated with MDRO infections after liver transplantation

Table 4 details the risk factors identified in LT recipients. According to the univariate analysis, factors associated with an increased risk of MDRO bloodstream infections included a history of preoperative ICU hospitalisation (OR = 55.12, 95% CI [10.74-282.94], *p* < 0.001), preoperative plasmapheresis (OR = 3.81, 95% CI [1.03-14.14], *p* = 0.046), a history of antimicrobial drug use within 30 days before surgery (OR = 11.50, 95% CI [3.72-35.54], *p* < 0.001), postoperative intestinal leakage (OR = 16.38, 95% CI [1.92-139.69], *p* = 0.011), abdominal paracentesis (OR = 6.46, 95% CI [2.28- 18.29], *p* < 0.001), a preoperative INR ≥ 2 (OR =   8.36, 95% CI [2.56-27.25], *p* < 0.001), and a preoperative creatinine concentration ≥ 120 mmol/L (OR = 5.62, 95% CI [2.00-15.79], *p* = 0.001). According to multivariate analysis, only a history of preoperative ICU stay (OR = 43.44, 95% CI [5.33-353.95], *p* < 0.001) and a preoperative INR ≥ 2 (OR = 7.07, 95% CI [1.01-49.26], *p* = 0.048) remained significant.

**Table 4.** Regression analysis of preoperative, intraoperative, and postoperative parameters and risk factors for multidrug-resistant bacterial bloodstream infections in liver transplant recipients.

	Univariate analysis			Multivariate analysis		
	Beta	OR (95%CI)	*p*	Beta	OR (95%CI)	*p*
Sex (male)	−0.3	0.74 (0.26–2.10)	0.572			
Age (years) ≥ 70	0.89	2.42 (0.38–15.49)	0.350			
BMI	−0.01	0.99 (0.87–1.12)	0.875			
Comorbidities						
Diabetes	−1.71	0.18 (0.02–1.56)	0.120			
Hypertension	0.37	1.45 (0.37–5.67)	0.593			
Hypertension and diabetes	1.47	4.35 (0.42–44.88)	0.217			
Ascites	−0.07	0.94 (0.36–2.40)	0.890			
Hepatic.encephalopathy	−0.94	0.39 (0.10–1.57)	0.186			
History of preoperative ICU hospitalisation	4.01	55.12 (10.74–282.94)	<.001	3.77	43.44 (5.33–353.95)	<.001
Preoperative history of open abdominal surgery	0.89	2.44 (0.90–6.63)	0.081			
Hospitalisation frequency in 1 year before surgery ≥ 10	18.31	89141511.57 (0.00–Inf)	0.990			
Plasma exchange	1.34	3.81 (1.03–14.14)	0.046	−0.21	0.81 (0.08–8.72)	0.865
Antibacterial drug use within 30 days before LT	2.44	11.50 (3.72–35.54)	<.001	1.03	2.81 (0.47–16.81)	0.257
Time of operationdayu ≥ 8h	−0.47	0.63 (0.14–2.73)	0.532			
The intraoperative blood loss ≥ 2L	0.49	1.62 (0.43–6.20)	0.478			
The intraoperative Urine output ml	0	1.00 (1.00–1.00)	0.076			
Length of ICU stay days	0.03	1.03 (0.99–1.08)	0.162			
Ventilator support time (h)	0	1.00 (1.00–1.01)	0.233			
CRRT	0.77	2.17 (0.81–5.82)	0.125			
Biliary complications						
Bile leakage	1.33	3.79 (0.64–22.61)	0.144			
Narrow	0.64	1.89 (0.54–6.68)	0.320			
Intestinal leakage	2.8	16.38 (1.92–139.69)	0.011	1.99	7.30 (0.41–130.61)	0.177
Abdominal paracentesis	1.87	6.46 (2.28–18.29)	<.001	1.27	3.58 (0.63–20.34)	0.151
Transplantation or open laparotomy again	0.71	2.02 (0.68–6.07)	0.208			
Splenectomy	17.13	27546394.98 (0.00–Inf)	0.989			
Acute rejection reaction	0.49	1.62 (0.43–6.20)	0.478			
Combined transplantation	16.02	9047991.64 (0.00–Inf)	0.991			
K+	−0.1	0.90 (0.34–2.38)	0.836			
Neutrophil ratio	0	1.00 (0.97–1.03)	0.866			
Prothrombin time (s) ≥ 20	0.96	2.60 (0.99–6.82)	0.052			
International normalised ratio ≥ 2	2.12	8.36 (2.56–27.25)	<.001	1.96	7.07 (1.01–49.26)	0.048
Total bilirubin (μmol/L) ≥ 122	−0.77	0.46 (0.18–1.21)	0.117			
Albumin (g/L) < 35	−0.59	0.55 (0.21–1.45)	0.228			
Creatinine (mmol/L) ≥ 120	1.73	5.62 (2.00–15.79)	0.001	1.04	2.82 (0.47–17.06)	0.259
Ammonia (μmol/L) ≥ 80	−0.25	0.78 (0.25–2.42)	0.670			
Total length of hospital stay (day)	−0.01	0.99 (0.97–1.01)	0.438			

BMI: Body Mass Index; MDRO: Multidrug Resistant Organism; PCT: Procalcitonin; Lac: Lactic acid; WBC: White blood cell count; NLR: Neutrophil-to-lymphocyte ratio; CRRT: Continuous renal replacement therapy.

## Discussion

This study sheds light on the prevalence and impact of BSIs with MDROs after LT. A significant portion of the patients, 39.7%, developed postoperative BSIs with MDROs, while 60.3% had non-MDRO BSIs. This finding underscores the considerable risk of BSIs in this population, particularly in the early postoperative period (within 30 days). In our study, 87.7% of patients developed BSIs within 30 days postoperatively, a finding consistent with previous research (Park et al., 2020). Unlike prior studies that identified body mass index (BMI) as a risk factor for mortality after LT (Liu et al., 2022), our study revealed no correlation between BMI and postoperative MDRO infections. Recent studies have also not reported significant differences in long-term mortality among patients with a BMI > or <40 (Kaur et al., 2021).

The absence of fever in one-third of patients with BSIs, regardless of the presence of MDRO, suggests that fever alone may not be a reliable indicator of infection after transplantation. A total of 64.4% of the patients had a WBC count less than 10 × 10^9^/L, similar to the findings of a previous study (Yang et al., 2018), which reported a WBC count less than 14 × 10^9^/L in 61.7% of LT recipients with CRKP bloodstream infection. The WBC count may not be a sensitive marker in LT recipients with BSIs. Instead, a combination of PCT and CRP may better detect BSIs. The marked difference in CRP levels between the MDRO and non-MDRO groups suggests that CRP is potentially a more effective marker of infection severity, especially in MDRO patients (Ashkenazi-Hoffnung et al., 2016; Lubell et al., 2023). To define the predictive value of CRP more clearly in predicting MDRO infections after LT, larger-scale, multicenter studies are essential.

MDRO infections were associated with significantly worse postoperative outcomes, including longer ICU stays and greater rates of complications such as bowel leakage and abdominal paracentesis. The higher mortality rates at 28-day and 90-day intervals in the MDRO group emphasise the severity of these infections. The predominance of gram-negative bacilli in blood infections aligns with previous literature (Park et al., 2020), and the high rate of drug-resistant bacteria highlights the treatment challenges and the need for careful antibiotic stewardship.

In this study, 58.2% of the pathogens were gram-negative bacilli, with gram-positive cocci (32.7%) and fungi (9.1%) constituting a smaller proportion. These findings align with a national study that analysed 1,380 pathogen strains and reported a predominance of gram-negative bacilli (69.57%), while gram-positive bacilli and fungi accounted for 20.07% and 10.36%, respectively (Wu et al., 2015). However, several single-center studies have reported a greater incidence of gram-positive bacterial infections than gram-negative infections (Kawecki et al., 2014; Liu et al., 2011).

In our patient group, 54.5% had drug-resistant bacterial infections, 31.8% of which were identified as multidrug-resistant. The most common source of these infections was the abdominal region (21.3%), followed by infections related to intravascular catheters (20.4%). This finding is consistent with the findings of a previous study and can be associated with the complex nature of LT and the routine practice of maintaining postoperative drainage tubes (H. K. Kim et al., 2013). These complications are among the leading causes of hospital mortality. Central venous cannulation, a critical part of ICU care after LT for fluid infusion, parenteral nutrition, and haemodynamic monitoring, also carries inherent risks due to its invasive nature. Catheter-associated bloodstream infections are among the most severe complications of central venous catheterisation, and significantly impact treatment costs and long-term patient outcomes. International studies have documented catheter-associated infection rates ranging from 6.3% to 23% of all hospital-acquired bacteraemia (Horan et al., 2008). The absence of intravascular catheters in place for 12–24 h after transplantation has been shown to result in an infection rate of 5.9%, which more than doubles if the catheter remains for more than 24 hours (Tan et al., 2014). Therefore, early catheter removal after surgery is recommended to minimise the risk of related adverse events.

Our study revealed significant correlations between various preoperative and postoperative factors and the occurrence of MDRO bloodstream infections after LT. The key risk factors included a history of ICU hospitalisation before transplantation, more than ten hospitalisations in the year preceding the surgery, antibiotic use within 30 days before transplantation, a preoperative INR > 2, and a preoperative creatinine > 120 mmol/L. Postoperative risk factors such as bowel leakage, laparotomy, and splenectomy were also associated with MDRO infections. Logistic regression analyses reinforced these findings, particularly highlighting the importance of preoperative ICU stay, plasma exchange, antibiotic use, intestinal leakage, abdominoperineal paracentesis, elevated preoperative INR, and creatinine levels in developing MDRO infections after transplant. In particular, the study revealed a substantially greater 90-day mortality rate among MDRO bloodstream infection recipients than among those without (13.7% vs. 2.7%) (Duan et al., 2019; Jia et al., 2020; Mawatari et al., 2018; Ogawa et al., 2012; Sacleux & Samuel, 2019; Tumbarello et al., 2014; Wang et al., 2019; Wang et al., 2023; Yao et al., 2019).

These correlations agree with the literature. Preoperative ICU stays, often involving critically ill patients with multiple organ dysfunctions and underlying diseases, are known to increase susceptibility to haematologic system infections, particularly MDRO (Liu et al., 2011). The INR, a vital measure of coagulation in end-stage liver disease patients, is directly related to patient prognosis, and higher preoperative INR is a known risk factor for postoperative mortality (Mawatari et al., 2018; Sacleux & Samuel, 2019; Yao et al., 2019). An INR > 2 has been significantly associated with 28-day mortality in adult sepsis patients (Wang et al., 2023), indicating that a higher INR before surgery might contribute to increased susceptibility to MDRO infections, possibly due to poorer hepatic function and increased risk of renal and lung injury (Ogawa et al., 2012).

This study highlights the complexities of managing BSIs in LT recipients, especially with the growing challenge of MDROs. This finding underscores the need for rigorous infection control, prompt and appropriate antimicrobial therapy, and continuous monitoring. The importance of developing targeted prophylactic and therapeutic interventions for this high-risk population is evident, as is the need for future research focusing on treatment optimisation and novel strategies against MDROs in transplant medicine (Ashkenazi-Hoffnung et al., 2016; Park et al., 2020).

This study has several limitations. First, this was a single-center retrospective study with potential regional biases in pathogen epidemiology. Second, we could not assess preoperative nasal colonisation by pathogenic bacteria, making the impact of bacterial carriers on post-transplant MDRO bloodstream infections unclear. The relationship between postoperative immunosuppressive management and MDRO infection is unclear, possibly due to limited sample size. Third, sample size calculation was not performed due to limited transplant cases. Future research should aim for larger, multicenter studies to develop a predictive model for MDRO bloodstream infections after transplant, which could significantly improve long-term outcomes for transplant recipients.

## Conclusion

Multidrug-resistant bacterial bloodstream infections after liver transplantation tend to occur early in the postoperative period (<30 days) and are associated with high mortality and a lack of specific clinical manifestations. A history of preoperative intensive care unit (ICU) hospitalisation and an international normalised ratio (INR) > 2 may be risk factors for multidrug-resistant bacterial bloodstream infections after liver transplantation.

## Data Availability

The datasets used in this study can be obtained from the corresponding authors upon reasonable request.
